# Chronic Pancreas Allograft Rejection Followed by Successful HLA-Incompatible Islet Alloautotransplantation: A Novel Strategy?

**DOI:** 10.3389/ti.2023.11505

**Published:** 2023-08-24

**Authors:** Denise M. J. Veltkamp, Michiel F. Nijhoff, Dennis A. J. van den Broek, Maren Buntinx, Jesper Kers, Marten A. Engelse, Volkert A. L. Huurman, Dave L. Roelen, Sebastiaan Heidt, Ian P. J. Alwayn, Eelco J. P. de Koning, Aiko P. J. de Vries

**Affiliations:** ^1^ Division of Nephrology, Department of Medicine, Leiden University Medical Center, Leiden, Netherlands; ^2^ Leiden Transplant Center, Leiden University Medical Center, Leiden, Netherlands; ^3^ Division of Endocrinology and Metabolism, Department of Medicine, Leiden University Medical Center Leiden, Leiden, Netherlands; ^4^ Department of Dermatology, Leiden University Medical Center, Leiden, Netherlands; ^5^ Department of Pathology, Leiden University Medical Center, Leiden, Netherlands; ^6^ Department of Transplant Surgery, Leiden University Medical Center, Leiden, Netherlands; ^7^ Department of Immunology, Leiden University Medical Center, Leiden, Netherlands

**Keywords:** antibody-mediated rejection, donor specific antigen (DSA), islet transplantation, re-transplantation, simultaneous kidney pancreas transplantation

## Abstract

The purpose of pancreas or islet transplantation is to restore glycemic control in order to mitigate diabetes-related complications and prevent severe hypoglycemia. Complications from chronic pancreas allograft rejection may lead to transplantectomy, even when the endocrine function remains preserved. We present first evidence of a successful HLA incompatible islet re-transplantation with islets isolated from a rejecting pancreas allograft after simultaneous kidney pancreas transplantation. The pancreas allograft was removed because of progressively painful pancreatic panniculitis from clinically uncontrolled chronic rejection. The endocrine function was preserved. Induction treatment for this “islet alloautotransplantation” consisted of plasmapheresis, IVIg and alemtuzumab. At 1 year, the patient retained islet graft function with good glycemic control and absence of severe hypoglycemia, despite persistent low-grade HLA donor-specific antibodies. His panniculitis had resolved completely. In our point of view, islet alloautotransplantation derived from a chronically rejecting pancreas allograft is a potential option to salvage (partial) islet function, despite preformed donor-specific antibodies, in order to maintain stable glycemic control. Thereby it protects against severe hypoglycemia, and it potentially mitigates kidney graft dysfunction and other diabetes-related complications in patients with continued need for immunosuppression and who are otherwise difficult to retransplant.

## Introduction

The purpose of pancreas or islet transplantation is to restore glycemic control in order to mitigate diabetes-related complications and prevent severe hypoglycemia. Whole pancreas transplantation has become a successful strategy since the 1960s in patients with type 1 diabetes in need of a kidney transplantation, or who otherwise have life-threatening glycemic control to warrant the impact of maintenance immunosuppression [1].

However, long-term outcomes after simultaneous pancreas-kidney (SPK) transplantation have not improved evidently in past decades, amongst others due to chronic rejection [2, 3]. Antibody-mediated rejection (ABMR) is increasingly recognized as a cause of chronic rejection in the setting of pancreas transplantation as well [3–5]. Chronic rejection is often refractory to anti-rejection therapy resulting in complications such as bleeding, duodenal perforation, and fistula/abscess formation. This may necessitate pancreatectomy despite preserved endocrine function, as pancreatic rejection is often more targeted to exocrine tissue, (micro)vasculature, or duodenum [6, 7]. Re-transplantation following sensitization becomes increasingly difficult, as pancreas allocation in the Eurotransplant area is not primarily based on HLA matching and patients are often no longer eligible (e.g., age >50–55 years, comorbidity), by the time a suitable offer becomes available, leaving them with labile diabetes again. Since the turn of the century, islet transplantation has become a less invasive alternative for whole pancreas transplantation with clinical merit to prevent severe hypoglycemia in labile diabetes as well as improved metabolic control compared to an optimized insulin regimen, especially in patients who are already on maintenance immunosuppression [8]. We previously described the option of alloautotransplantation, a strategy to preserve endogenous insulin production by isolating and re-transplanting the islets after an allograft pancreatectomy [7]. However, evidence for HLA incompatible islet alloautotransplantation from a chronically rejecting pancreas transplant has not been described before to our knowledge.

Here we present the 1 year results of an HLA incompatible islet re-transplantation after graft pancreatectomy in a patient with progressively painful panniculitis from a clinically uncontrolled chronic rejection of the pancreas (and kidney) allograft with preserved endocrine function.

## Evidence

The evidence concerns a non-sensitized (virtual panel-reactive antibody; vPRA 0%) 50 year-old cytomegalovirus (CMV) seronegative male with a medical history of poorly controlled type 1b diabetes (T1D) and end-stage renal disease. The patient was diagnosed with type 1 diabetes at 8 years of age after a presentation of ketoacidosis. He was negative for anti-GAD, anti-IA-2 and anti-ZnT8 at time of referral and had undetectable C-peptide since childhood (<0.01 nmol/L). With a flexible four times daily insulin regimen (on average 0.8 units/kg daily), he suffered from recurrent hypoglycemia and poor glycemic control (HbA1c 90.3 mmol/mol Hb). The patient underwent SPK transplantation from an HLA-mismatched (2-2-2) CMV seronegative donor after cardiac death. The patient provided written consent for publication.

**FIGURE 1:**
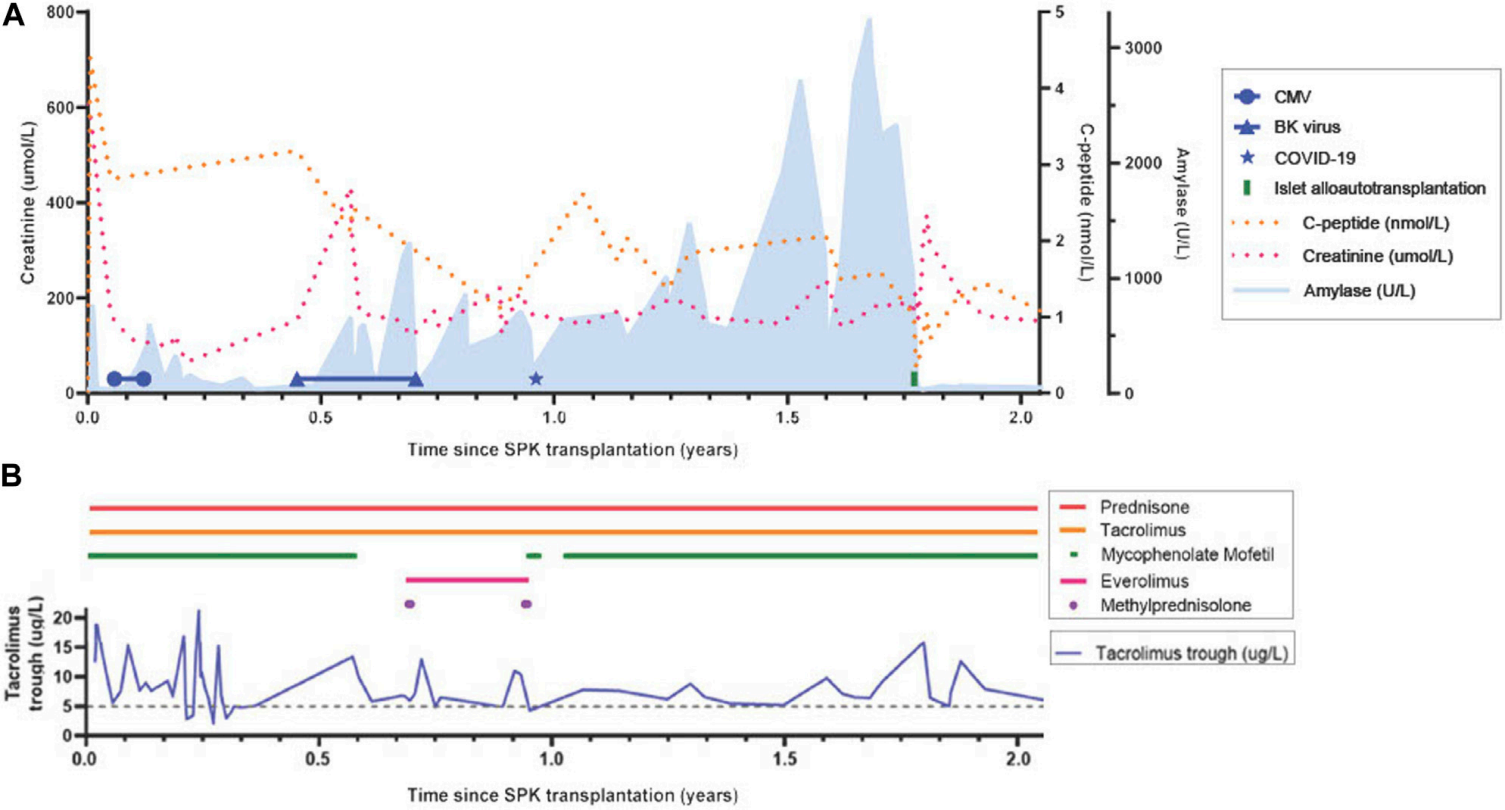
Timeline from simultaneous pancreas-kidney transplantation to 3 months post-islet alloautotransplantation. **(A)** Lab results: C-peptide, creatinine, amylase, BK-viremia, Cytomegaloviremia, COVID-19 infection. MFI DQB1*05:02-DQA1*01:02 (months since SPK): 997 (8); 943 (11); 871 (12); 841 (15); 1180 (24). Type 1 diabetes auto-antibodies remained negative. **(B)** Medication exposure: prednisone, tacrolimus and tacrolimus trough, mycophenolate mofetil, everolimus, methylprednisolone (1,000 mg, 3 days). Target tacrolimus trough level: 10–15 ng/mL within first 6 weeks, 5–8 ng/mL beyond 6 weeks; Target 12h-auc mycophenolate mofetil: 30–45 mg*h/L. CMV, Cytomegalovirus; SPK, simultaneous pancreas-kidney transplantation.

At SPK transplantation his immunosuppressive regimen consisted of alemtuzumab induction 30 mg s.c. and methylprednisolone 500 mg once, followed by tacrolimus 5 mg b.i.d., mycophenolate mofetil 750 mg b.i.d., and prednisolone 10 mg q.d. The postoperative course (Figure 1) was complicated, amongst others, by delayed renal allograft function and an unexpected primary CMV infection for which valganciclovir was initiated. Due to development of leukopenia and later BK-viremia, mycophenolate mofetil was discontinued and tacrolimus tapered.

**FIGURE 2:**
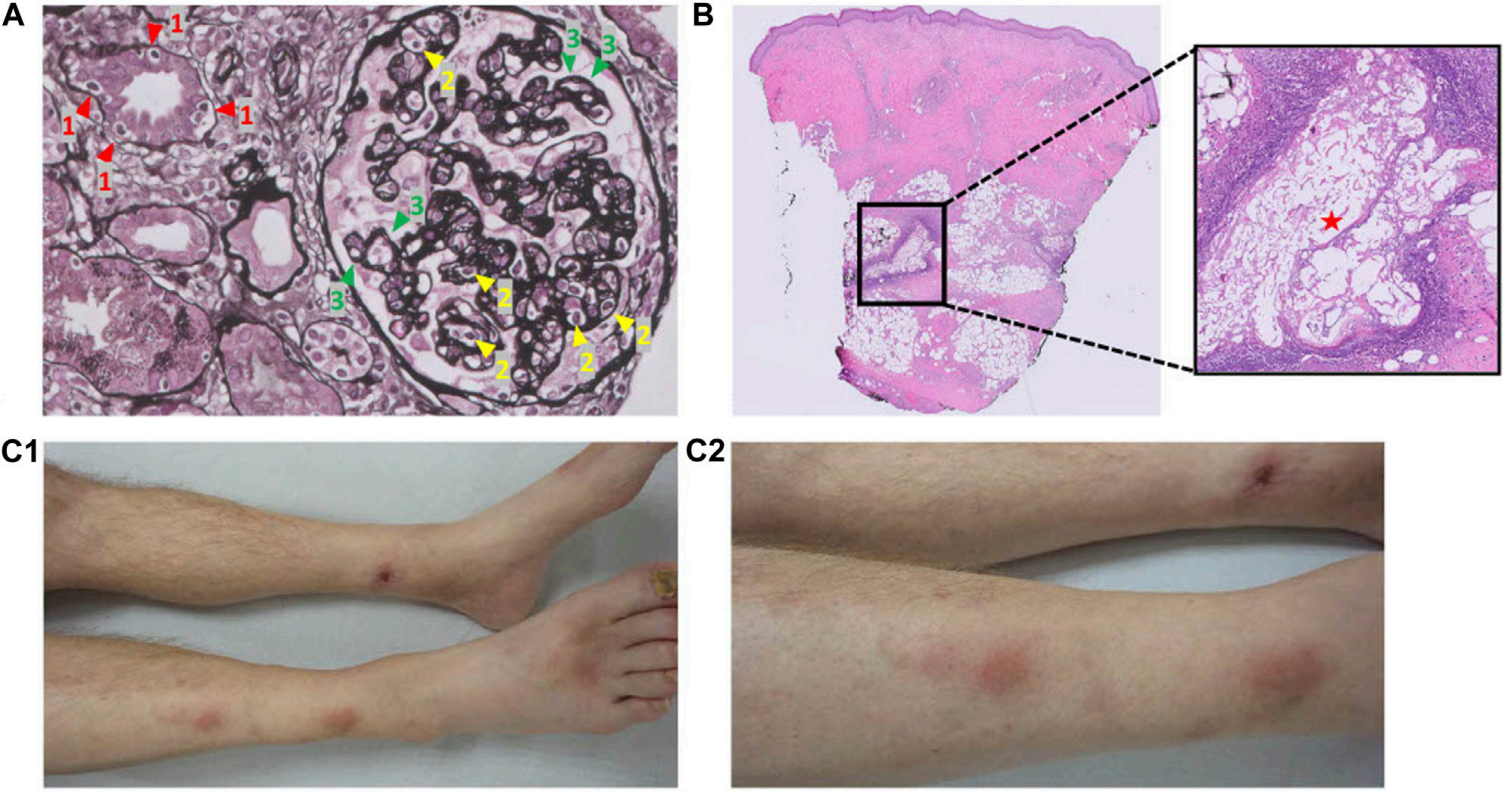
Pathology pictures of the kidney allograft biopsy and skin biopsy, and clinical pictures of the pancreatic panniculitis. **(A)** Kidney allograft biopsy: mixed-type chronic active T cell-mediated rejection and antibody-mediated rejection rich in plasma cells. 1 (red) = tubulitis; 2 (yellow) = glomerulitis; 3 (green) = double contours of the glomerular basement membrane. Banff: g2, i0, t1, v0, ptc1, mm0, cg2, i-IFTA2, t-IFTA2, ci2, ct2, cv1, ah1. **(B)** Skin biopsy: pancreatic panniculitis. Adipocytes have been saponified by the process of enzymatic fat necrosis, accompanied by neutrophilic inflammation. **(C)** Pancreatic panniculitis on both lower legs. **(C1)**: anterior view. **(C2)**: Lateral (right)/anterior view.

In the period from eight to 11 months posttransplant, two clinical episodes of pancreas rejection occurred each with right lower quadrant abdominal tenderness, graft pancreatitis on radiological imaging, a concomitant increase in serum amylase/lipase, and *de-novo* HLA donor-specific antibodies (DSAs) (immunodominant DQB1*05:02-DQA1*01:02 with low median fluorescence intensity (MFI) of 997 at 8 months and 943 at 11 months, and anti-DP4 with MFI 1800 at 11 months), measured with Luminex single antigen bead assays from LifeCodes (Immucor). Alternative causes of pancreatitis such as constipation, CMV relapse, and re-occurrence of T1D auto-antibodies were ruled out. A pancreas or, alternatively, a kidney allograft biopsy was not performed at first, as pancreas biopsy is not routine practice at our center due to experienced risk of complications, and kidney graft function initially remained stable. However, a kidney graft biopsy at 11 months, performed because of *de-novo* proteinuria (2.90 g/24 h), showed mixed-type chronic active T cell-mediated rejection (TCMR) and ABMR rich in plasma cells with negative staining for CMV, BK-virus (SV40), and C4d (Figure 2A). Both clinical rejection episodes were treated with methylprednisolone 1 g q.d. for 3 days only. However, second-line therapy (lymphocyte depleting antibodies) was withheld because of contraindications at that time (leukopenia and active viral complications: BK-viremia (8 months), and COVID-19 (11 months)). Pancreatectomy was considered but a watchful waiting strategy was chosen since endocrine function remained intact and no other complications were immediately evident. At 8 months, everolimus 0.75 mg b.i.d. was started next to tacrolimus to consolidate the first-line rejection treatment by pulsed steroids to control chronic rejection in the context of BK-virus reactivation and leukopenia. At 11 months, everolimus was substituted by mycophenolate mofetil because of proteinuria, which then improved. As complication of high-dose corticosteroids, vertebral compression fractures of the thoracic spine occurred.

At 20 months post SPK transplantation, progressively painful subcutaneous nodules on both lower legs developed (Figure 2C) necessitating opioid maintenance. A punch skin biopsy showed pancreatic panniculitis (Figure 2B), likely as complication of ongoing chronic pancreas allograft rejection, which has been described incidentally before in transplant recipients [9]. Since efficacy of second-line therapy is questionable for long-standing chronic (or mixed-type) rejection and because of progressive complications, pancreatectomy was deemed favorable over a third round of methylprednisolone or escalation with second-line therapy now that contraindications were absent [6]. Nonetheless, the pancreas’ endocrine function remained intact (HbA1c 38.5 mmol/mol, random C-peptide 1.6 nmol/L, glucose 5.4 mmol/L, without insulin therapy). As the patient was adamant to preserve his endocrine function 21 months after SPK transplantation, an islet alloautotransplantation was performed after pancreatectomy despite presence of (low-grade) DSAs. Before islet transplantation, a single round of plasma exchange was performed followed by intravenous immunoglobulin (IVIg) (1 g/kg). Alemtuzumab (15 mg s.c.) induction, methylprednisolone (500 mg day −1, 250 mg day 0, 125 mg day +1) and etanercept (50 mg s.c. day 0, 25 mg s.c. days 2/6/10) were prescribed following standard practice for allogeneic islet transplantation according to local protocol. At the operating room, directly after pancreatectomy the pancreas was flushed on the backtable using Ringer’s acetate solution supplemented with 5 mmol/L calcium at pH 7.35 prior to static cold storage transport to the islet isolation laboratory. Islet isolation was performed according to previously published protocols [10–14]. Macroscopically, the pancreas looked edematous but non-necrotic. With iodine and penicillin-streptomycin solution the pancreas was decontaminated. The pancreas was infused with 1 vial (2533 IU) collagenase solution combined with one vial of neutral protease solution (200 IU) (both Serva NB-1® (Serva Electrophoresis GmbH, Heidelberg, Germany) in Ringer’s acetate solution supplemented with 5 mmol/L calcium at pH 7.35. Gentamycin, ciprofloxacin, vancomycin and amphotericin B were added to all fluids in the isolation’s procedure. In the final product, both the gram staining and the endotoxin test were negative. The islets were re-transplanted intraportally directly after the isolation procedure. The alloautograft contained 313000 Islet equivalents (IEQ) (4013 IEQ/kg patient) in a volume of 12 mL with a purity of 6%. Strict glycemic control was maintained by continuous intravenous insulin (glucose target 4–7 mmol/L) for 48 h, after which s.c. insulin was recommenced to maintain the tight glycemic target.

At 15 weeks after this islet alloautotransplantation, the patient was treated with 8 units of long-acting insulin once daily. Time-in-range of glucose concentrations was 96% (3.9–10 mmol/L; as measured by Flash Glucose Monitoring) with an HbA1c of 42.7 mmol/mol. Maximum C-peptide was 2.32 nmol/L (maximum glucose 12.4 mmol/L) during a 2 h mixed-meal tolerance test (MMTT). One year after the islet alloautotransplantation the patient retained islet graft function with absence of severe hypoglycemia. At that time, he used metformin/dapagliflozin 850mg b.i.d. combined with 20 units of long-acting insulin a day, with which he had a time-in-range of 80%–90% and an estimated HbA1c of 48 mmol/mol. Baseline C-peptide was 0.30 nmol/L with glucose of 4.1 mmol/L, and rose to 1.01 nmol/L with a maximum glucose of 9.1 mmol/L during the MMTT. Together, this amounts to clinical treatment success as defined by a “good” function according to the Igls criteria, a beta score of 5, and a BETA-2 score of 13.55 [15]. Anti-GAD, anti-IA-2 and anti-ZnT8 remained negative and kidney function clinically stable (creatinine 134 umol/L with an albumin/creatinine ratio of 30.3 mg/mmol). The panniculitis recovered completely. The preformed immunodominant DSA DQB1*05:02-DQA1*01:02 remained present at 1 year with a MFI of 1180. Anti-DP4 remained undetectable after the islet alloautotransplantation.

## Discussion

This report describes 1 year evidence of a successful (according to the Igls criteria) islet alloautotransplantation in a patient with preformed HLA-DSAs after allograft pancreatectomy for likely chronic rejection of the pancreas. Although we did not obtain histological evidence, the fact that a clinical syndrome of pancreatitis occurred twice (localized pain, rise in pancreatic enzymes, and edematous pancreas at imaging) after a period of immunosuppressive underexposure with development of a dnDSA made a clinical diagnosis of pancreas rejection likely. Non-alloimmune causes of pancreatitis were excluded albeit that the unexpected primary CMV infection may have contributed since CMV has been associated with heterologous immunity [16]. After reconfirming seronegativity of donor and recipient, it remains unknown how the CMV infection occurred. A kidney biopsy during the second episode of pancreatitis showed mixed chronic-active TCMR and ABMR further corroborating the likelihood of rejection.

Although some discordancy for rejection (12.5% kidney-only rejections) has been observed by Parajuli et al., that study did not stratify for clinical context such as simultaneous pancreas dysfunction and presence of DSA [17]. A study by Uva et al. showed that a positive renal biopsy for rejection correctly predicts pancreatic rejection in 86% of cases with concurrent pancreatic dysfunction, albeit they also did not stratify by presence of DSA [18]. Furthermore, there is debate whether discordancy truly exists or whether it’s merely a matter of time [19, 20]. Our report illustrates the timely development from a single episode of clinical pancreatitis with DSA formation to recurrent pancreatitis, to eventually kidney transplant dysfunction with biopsy proven chronic mixed type ABMR and a biopsy-proven panniculitis to underscore the likelihood of chronic pancreas rejection. At the time, active viral complications and leukopenia contraindicated escalation to second-line therapy, such as ATG or alemtuzumab, to have improved rejection control [21].Notably, the pancreas graft’s endocrine capacity appeared relatively unaffected by the pancreatitis episodes perhaps also questioning the likelihood of rejection. However, distinctive rejection patterns of pancreatic endocrine and exocrine cells have been described before, yet the pathophysiological mechanism is not completely clear. It might be reflective of differential HLA expression [22]. Exocrine tissue and the pancreas (micro)vasculature are often the primary targets of rejection, whereas islets remain relatively unaffected. Pancreatitis from chronic (acinar) rejection might subsist by ongoing release of inflammatory danger signals, such as proteases, from continued pancreatitis. The persistent acinar damage and chronic vascular injury might trigger a progressive fibrogenic reaction that could eventually impair β-cell function [22]. The painful complication of the progressive pancreatic panniculitis, first described by Chiari in 1883, is not completely understood, but is thought to be subcutaneous fat necrosis from circulating proteases and enzymes released from an inflamed pancreas, which necessitated the use of opioids in our case [9, 23]. A pancreas transplantectomy and re-transplanting the islets, when endocrine function remains largely preserved, could theoretically break the vicious cycle. From our point of view, the precise etiology of the pancreatitis does not diminish the observation that it is possible to perform an islet alloautotransplantation in the context of a low-grade DSA in patients with limited access to transplantation. The patient’s vPRA had increased to 87% making him an unlikely candidate for pancreas re-transplantation at his age or for HLA compatible islet transplantation. Islets are thought not to express HLA class II, except under conditions of stress/inflammation [3]. We therefore removed present antibodies using plasma exchange and IVIg before islet alloautotransplantation in order to create a “window of opportunity before DSA recurrence” to infuse islets that could have upregulated HLA class II expression due to stress of isolation and culture, instant blood-mediated inflammatory reaction, or hypoxia. Islets are relatively protected from DSAs once vascularized due to endothelial chimerism and vascular sequestration of DSAs [24]. Notably, a recent single-center study, published after our case occurred, showed favorable outcomes of allogeneic islet transplantation in the presence of preformed DSAs, questioning in hindsight the necessity for pretransplant plasma exchange and IVIg [25]. Low-grade DSA convey excellent outcome [26].

Before deciding to perform the islet alloautotransplantation, we discussed whether to treat the likely pancreas allograft rejection with a third round of methylprednisolone and a postponed second-line treatment for chronic (or mixed-type) ABMR, which consists of alemtuzumab, plasma exchange, and/or IVIg at our center. Viral complications (BK-viremia, COVID-19) had prohibited escalation before. However, we felt that efficacy of such therapy for longer-standing chronic active perhaps antibody-mediated pancreas rejection would be questionable and that, given the progressive complications (panniculitis) necessitating chronic use of opioids, a more definite solution was warranted. The persistent immunodominant HLA-DSA DQB1*05:02-DQA1*01:02 at 1 year post-islet alloautotransplantation suggests indeed that second-line treatment would not have made the DSA disappear. Although speculative, we also hypothesized that taking away the subsisting inflammatory stimulus (exocrine tissue rejection/pancreatitis) would likely increase the chance of a more sustained response to novel induction treatment and better preserve his kidney function considering his highly-immunized status [27]. A limitation of this point of view is that an auto-immune etiology of his type-1 diabetes could not be established (no detectable auto-antibodies), which might have contributed to a more favorable outcome of the islet transplantation.

## Conclusion

Successful islet alloautotransplantation from a likely chronically rejecting pancreas with preserved endocrine function is possible to salvage (partial) β-cell function after pancreatectomy. Preformed low-grade DSAs are not necessarily a contraindication. This strategy may be considered to prevent relapse of inadequate glycemic control with recurrent severe hypoglycemic events and have improved metabolic control with better preserved kidney transplant function, when there is ongoing need for immunosuppression and the patient is an unlikely candidate for pancreas re-transplantation.

## Data Availability

The original contributions presented in the study are included in the article/supplementary material, further inquiries can be directed to the corresponding author.

## Abbreviations

ABMR, Antibody-mediated rejection; CMV, Cytomegalovirus; C-peptide, Connecting peptide; DSA, Donor-specific antibody; HLA, Human leukocyte antigen; IEQ, Islet equivalent; IVIg, Intravenous immunoglobulin; MFI, Median fluorescence intensity; MMTT, Mixed-meal tolerance test; SPK, Simultaneous pancreas and kidney transplantation; T1D, Type 1 diabetes; TCMR, T cell-mediated rejection.
